# Impact of liver fat on the differential partitioning of hepatic triacylglycerol into VLDL subclasses on high and low sugar diets

**DOI:** 10.1042/CS20171208

**Published:** 2017-10-17

**Authors:** A. Margot Umpleby, Fariba Shojaee-Moradie, Barbara Fielding, Xuefei Li, Andrea Marino, Najlaa Alsini, Cheryl Isherwood, Nicola Jackson, Aryati Ahmad, Michael Stolinski, Julie A. Lovegrove, Sigurd Johnsen, A.S. Jeewaka R. Mendis, John Wright, Malgorzata E. Wilinska, Roman Hovorka, Jimmy D. Bell, E. Louise Thomas, Gary S. Frost, Bruce A. Griffin

**Affiliations:** 1 Faculty of Health and Medical Sciences, University of Surrey, Guildford, U.K.; 2 Faculty of Health Sciences, University Sultan Zainal Abidin, 21300 Kuala Nerus,Terengganu, Malaysia.; 3 Hugh Sinclair Unit of Human Nutrition and Institute for Cardiovascular and Metabolic Research, University of Reading, Reading, U.K.; 4 Diabetes Modelling Group, Institute of Metabolic Science, University of Cambridge, Cambridge, U.K.; 5 Metabolic and Molecular Imaging Group, MRC Clinical Sciences Centre, Imperial College London, Hammersmith Hospital, London, U.K.; 6 Research Centre for Optimal Health, Department of Life Sciences, University of Westminister, London, U.K.; 7 Nutrition and Dietetic Research Group, Section of Investigative Medicine, Division of Diabetes, Endocrinology and Metabolism, Imperial College London, U.K.

**Keywords:** non alcoholic fatty liver disease, plasma lipoproteins, Sugar, triglycerides

## Abstract

Dietary sugars are linked to the development of non-alcoholic fatty liver disease (NAFLD) and dyslipidaemia, but it is unknown if NAFLD itself influences the effects of sugars on plasma lipoproteins. To study this further, men with NAFLD (*n* = 11) and low liver fat ‘controls’ (*n* = 14) were fed two iso-energetic diets, high or low in sugars (26% or 6% total energy) for 12 weeks, in a randomised, cross-over design. Fasting plasma lipid and lipoprotein kinetics were measured after each diet by stable isotope trace-labelling.

There were significant differences in the production and catabolic rates of VLDL subclasses between men with NAFLD and controls, in response to the high and low sugar diets. Men with NAFLD had higher plasma concentrations of VLDL_1_-triacylglycerol (TAG) after the high (*P*<0.02) and low sugar (*P*<0.0002) diets, a lower VLDL_1_-TAG fractional catabolic rate after the high sugar diet (*P*<0.01), and a higher VLDL_1_-TAG production rate after the low sugar diet (*P*<0.01), relative to controls. An effect of the high sugar diet, was to channel hepatic TAG into a higher production of VLDL_1_-TAG (*P*<0.02) in the controls, but in contrast, a higher production of VLDL_2_-TAG (*P*<0.05) in NAFLD. These dietary effects on VLDL subclass kinetics could be explained, in part, by differences in the contribution of fatty acids from intra-hepatic stores, and de novo lipogenesis. The present study provides new evidence that liver fat accumulation leads to a differential partitioning of hepatic TAG into large and small VLDL subclasses, in response to high and low intakes of sugars.

## Introduction

Non-alcoholic fatty liver disease (NAFLD) is a common condition, defined histologically, as an excess of macro-vesicular steatosis (>5%) in the absence of a high intake of alcohol [1]. In addition to being a progenitor of end-terminal liver diseases, NAFLD has been linked to the metabolic syndrome, and is a potential source of elevated plasma triacylglycerol (TAG) and abnormalities in plasma lipoproteins, known as an atherogenic lipoprotein phenotype (ALP) [2–4].

Elevated plasma TAG promotes the development of an ALP through the extra-cellular remodelling of plasma low and high density lipoproteins (LDL and HDL) into small, dense particles with increased potential to promote atherosclerosis [5]. Plasma TAG may be raised by an overproduction of its principal transporter VLDL in the liver, and/or impaired clearance of VLDL from the plasma [6]. VLDL is secreted from the liver as a spectrum of particles that vary in size, composition and metabolic properties, which can be subdivided on the basis of hydrated density into two discrete subclasses of large, TAG-rich VLDL_1_ and smaller VLDL_2_ (Svedberg flotation units (Sf) of 60–400 and 20–60, respectively) [7]. The particle size of VLDL in the liver in the fasted, post-absorptive state is largely determined by the availability of lipid in the form of non-esterifed fatty acids (NEFA) from peripheral adipose tissue (systemic source) or splanchnic sources, the latter of which includes visceral adipose tissue, intra-hepatic stores, and synthesis of fatty acids by de novo lipogenesis (DNL) in the liver [8].

Free sugars in food and sugar-sweetened beverages have been implicated in the development of dyslipidaemia and NAFLD, either through the direct lipogenic effects of sugars on liver fat and VLDL metabolism, and/or via the indirect effects of the energy from sugars on body weight [9]. While DNL makes a relatively small contribution to VLDL-TAG production, this has been shown to increase substantially when a high proportion of dietary energy is supplied as sugars, especially sucrose and fructose (>20% total energy) [10]. However, the extent to which liver fat affects the handling of hepatic fatty acids and alters VLDL metabolism in response to intakes of sugars representative of a Western diet, is currently unknown. The present study tested the hypothesis that liver fat influences the metabolic effects of a high relative to a low intake of sugars (representative of the upper and lower 2.5th percentiles of intake in the UK), on plasma lipoproteins, by altering the kinetics and source of fatty acids for the production of VLDL subclasses.

## Materials and methods

### Participants

Participants were men (aged 40–65 years, BMI 25–30) at increased cardio-metabolic risk, as determined by a 1 to 10 risk score used previously in the ‘*RISCK’* study [11]. Men with a cardio-metabolic score of ≥4 and *APO ε3/ε3* genotype (to exclude possible confounding effects of different apo E isoforms on lipid metabolism), underwent an assessment of intra-hepatocellular lipid (IHCL) by magnetic resonance spectroscopy (MRS) for assignment to a group with NAFLD (>5.56% IHCL, *n* = 11) or low liver fat (controls) (<5.56% IHCL, *n* = 14) [12]. Exclusion criteria included diabetes and any medical condition other than NAFLD, lipid-lowering medication, unstable weight in the preceding 3 months, and an intake of alcohol exceeding 20 g/day. All participants provided written informed consent before taking part in the study, which received favourable ethical opinions from Surrey Research Ethics Committee (Ref. 08/H1109/227), and the University of Surrey's Ethics Committee (Ref. EC/2009/29). The trial was registered on Clinical Trials.gov (Ref. NCT01790984).

### Study design

The study had a randomised, two-way cross-over design, with two 12 weeks dietary interventions. After an initial 4 weeks run-in period on their habitual diet, participants were randomised to either a high or low sugar diet, using a computer-generated sequence of treatments in sealed envelopes. The two diets were iso-energetic and contained the same macronutrient composition. Participants returned to their habitual diet for 4 weeks washout, before crossing-over to the alternative diet for a further 12 weeks. During the dietary interventions, participants were instructed to maintain their habitual level of physical activity. Certain outcome measures were determined before and after each diet (body weight, percentage body fat, plasma lipids, glucose and serum insulin), while others were measured at the end of each diet (stable-isotope tracer kinetics, lipoprotein composition, IHCL and body fat distribution by MRS).

### Dietary interventions

Intakes of total carbohydrates and sugars were based on mean intakes for men aged 40–65 years in the UK's National Diet & Nutrition Survey (NDNS), with target intakes for non-milk extrinsic sugars (NMES) on the high and low sugar diets corresponding to the upper and lower 2.5th percentile of intake in the UK population, respectively [13]. The term NMES, as originally defined by the UK's Department of Health [14], included free sugars added to food (including 50% of sugars in tinned and dried fruit), but excluded sugars in whole fruit, and lactose, primarily from cows’ milk [15]. The content of sugars in the two diets was achieved by a dietary exchange of sugars for starch using a range of commercially available foods, as described in the Supplementary Material. Dietary intakes were assessed by the completion of 3-day diet diaries during the final week of each dietary intervention (2 weekdays and 1 weekend day). Diaries were analysed by a single operator using *DietPlan 6* (version 6.50, Forestfield Software Ltd, UK).

### Metabolic study (Post-diets)

The study protocol is shown in Supplementary Figure S1. The evening before the metabolic study, participants consumed a set volume of deuterated water (^2^H_2_O 3 g/kg body water; 50% after a standardised low fat, low fibre meal (1900 h) and 50% 3 h later at 2200 h). They then fasted and drank only water enriched with ^2^H_2_O (4.5 g ^2^H_2_O/l drinking water). The following morning, a blood sample was taken to measure deuterium enrichment of palmitate in VLDL_1_ and VLDL_2_-TAG, and plasma water to measure DNL (for calculation see the Supplementary Material). A primed, 10 h constant iv [1-^13^C]leucine infusion (1 mg/kg; 1 mg/(kg h)) (99%, Cambridge Isotopes) was administered to measure VLDL_1_, VLDL_2_, intermediate density lipoprotein (IDL), LDL_2_ and LDL_3_-apoprotein B (apoB) kinetics. An 8 h constant iv infusion of [U-^13^C]palmitate (99%, Cambridge Isotopes) bound to human albumin (5%, 0.01 µmol/(kg min)), was administered to measure palmitate production rate (PR, assumed to be mainly from systemic adipose tissue lipolysis), and the percentage contribution of systemic NEFA to the export of TAG in VLDL_1_ and VLDL_2_. An intravenous bolus of [1,1,2,3,3-^2^H_5_]glycerol (75 µmol/kg) (99%, Cambridge Isotopes) was administered to measure VLDL_1_ and VLDL_2_-TAG PR and fractional catabolic rate (FCR). Blood samples were taken at sequential time intervals to measure the isotopic enrichment and concentrations of plasma palmitate, αketoisocaproate (αKIC) and glycerol, and the enrichment and concentrations of apoB, TAG-palmitate and TAG-glycerol in the lipoprotein fractions, as reported previously [16,17]. At the end of each dietary intervention period, the activity of lipoprotein lipase (LPL) and hepatic lipase (HL) in plasma was measured before and 15 min after an intravenous injection of 50 U/kg heparin, as previously described [18].

### Magnetic resonance imaging and spectroscopy

Whole body magnetic resonance imaging (MRI) scans were obtained on a 1.5T Phillips Achieva system (Philips Medical Systems, Best, The Netherlands). Volumes of intra-abdominal and subcutaneous abdominal adipose tissue were calculated from the abdominal region between the slices containing the bottom of the lungs/top of the liver, and femoral heads. Spectra were analysed by a single trained observer (ELT) using AMARES. Liver fat (IHCL) was measured relative to liver water content, as described previously [19]. Seventeen of the 25 participants who completed both diets, underwent a post-dietary analysis of IHCL and body fat distribution by MRS.

### Laboratory methods

VLDL_1_, VLDL_2_, IDL, LDL_2_ and LDL_3_ were separated by sequential ultracentrifugation [7]. Plasma TAG, VLDL_1_ and VLDL_2_-TAG were extracted, and the isotopic enrichment of glycerol and TAG-palmitate in these extracts was measured by gas chromatography mass spectrometry (GCMS), as described previously [16]. The isotopic enrichment of leucine in VLDL_1_, VLDL_2_ and IDL-apoB, and plasma αKIC enrichment, was measured by GCMS [17]. Leucine enrichment in LDL_2_ and LDL_3_-apoB was measured as the *N-*acetyl, *n*-propyl-ester derivative and analysed by GC-combustion isotope ratio MS (Delta plus XP isotope ratio MS, Thermo Scientific). Plasma ^2^H_2_O enrichment was measured with a Gasbench II inlet system and isotope ratio MS using platinum catalyst rods to liberate hydrogen gas. Isotopic enrichment was measured relative to laboratory standards, which had been previously calibrated against international standards; Vienna Standard Mean Ocean Water and Standard Light Arctic Precipitation (International Atomic Energy Agency, Vienna, Austria). LPL and HL were measured in post-heparin plasma by the Confluolip Lipase test (Progen Biotechnik, Heidelberg). Plasma NEFA, total cholesterol, TAG, lipoprotein fraction TAG and cholesterol were measured by enzymatic assays using a Cobas MIRA (Roche, Welwyn Garden City, UK). Apolipoprotein B (apo B) in lipoprotein fractions was measured by an in-house ELISA. Plasma apolipoproteins C-II, C-III and E were measured by commercially available ELISAs (Biomedica, GmbH & Co, Wien, Austria), and small dense (sd) LDL-cholesterol by a precipitation method (Randox Laboratories Ltd) on an ILab 650 (Werfen). *APO E* genotype was determined by quantitative polymerase chain reaction and Southern blotting.

### Data analysis

Tracer enrichment of αKIC, leucine, palmitate and glycerol was expressed as tracer/tracee ratio (TTR) corrected for baseline enrichment. Lipoprotein kinetics were analysed by compartmental modelling, as described previously [16,17]. These models and the calculation of the fatty acid contribution to VLDL-TAG PR, together with further details of the methods, are described in the Supplementary Material.

### Statistical methods

Data are expressed as means (± SEM) for normally distributed variables, and log_10_ transformed geometric means for non-normally distributed variables.

*Statistical modelling*. Data are expressed as estimates of contrasts of least squares means for normally distributed variables, and as ratios of geometric means for logarithmically transformed variables. For outcome measures for which there were four samples from each participant (pre- and post-diets, for each period), the post-diet measurements (NAFLD and controls for the 2-period cross-over, logarithmically transformed or not, as appropriate), were analysed as dependent variables in a general linear mixed model, with the following fixed categorical, non-random, explanatory effects: period, treatment (low and high sugar diet), period by treatment interaction (to detect carry-over effects), liver fat level (NAFLD and control) and treatment by liver fat level interaction. The pre-diet measurements (logarithmically transformed or not, as appropriate) for each period, and body weights (pre- and post-diets) were included as covariates in the model, with participant as a model random effect. For outcome measures for which there were two samples for each participant (post-diets; end of each dietary intervention period only), each measurement for the combined groups (NAFLD and controls), for the 2-period cross-over, were analysed in a general linear mixed model with the same fixed categorical effects as above, and body weights (pre- and post-diets) as covariates. Variables for which there was no significant carry-over effect, were modelled as above, omitting the period-by-treatment interaction from the model. There was only evidence of significant treatment by period interactions (carry-over effect) at the 5% level for VLDL_1_-TAG FCR, which was modelled using data from the first period only. Modelling was performed using procedure MIXED of SAS Version 9.2 (SAS Institute, Cary, NC, USA).

## Results

Twenty-five men completed the study. The baseline characteristics of the NAFLD and control groups, including age, body weight, BMI, waist circumference and biochemical measures, were similar, except for plasma TAG, which was 42% higher in men with NAFLD than controls (*P*<0.05, Table 1).

**Table 1:** Group characteristics at baseline

	NAFLD (*n* = 11)	Controls (*n* = 14)
Age, years (range)	59 (49–64)	54 (41–65)
Body weight (kg)	90.0 ± 2.2 (75.6–102.4)	89.7 ± 2.4 (78.3–107.9)
BMI (kg/m^2^)	28.9 ± 0.3 (26.9–30.8)	28.4 ± 0.5 (26.0–31.0)
Waist circumference (cm)	104 ± 2 (93–113)	104 ± 1 (100–114)
Liver fat (%)	17.2 ± 2.7^1^ (7.9–36.8)	2.5 ± 0.3 (0.5–4.6)
Plasma TAG (mmol/l)	1.89 ± 0.27^2^ (1.10–4.01)	1.33 ± 0.23 (0.60–3.80)
Plasma cholesterol (mmol/l)	5.91 ± 0.25 (4.60–7.20)	5.51 ± 0.28 (4.30–7.20)
Plasma HDL cholesterol (mmol/l)	1.22 ± 0.08 (1.00–2.00)	1.24 ± 0.08 (0.90–2.10)
Plasma glucose (mmol/l)	5.73 ± 0.11 (4.90–6.10)	5.46 ± 0.12 (4.90–6.40)
Systolic BP (mmHg)	131 ± 7 (113–177)	134 ± 3 (110–156)
Diastolic BP (mmHg)	86 ± 4.5 (67–113)	84 ± 2.7 (62–93)

Values are means ± SEM (ranges). Significant difference between groups: ^1^*P*<0.001; ^2^*P*<0.05.

### Dietary intake and changes in body weight

Self-recorded dietary intakes were monitored by regular visits to the homes of participants, and indicated that dietary compliance was maintained. There was no difference in reported energy intake between diets, or differences in energy intake, macronutrients or alcohol between NAFLD and controls on either diets (Supplementary Table S1). The high sugar diet resulted in a higher intake of total sugars and NMES (26% total energy) in comparison with the baseline and low sugar diet (6% total energy) in both men with NAFLD and controls (*P<*0.01 for all comparisons). The high sugar diet was also lower in starch (*P*<0.01) than the low sugar diet in both groups. Percent energy intake from dietary fat was significantly lower on the high sugar diet in controls (*P*<0.001).

Body weight was higher after the high versus low sugar diet in NAFLD (*P<*0.001) and controls (*P*<0.01), with both groups gaining and losing approximately 2 kg on the high and low sugar diets, respectively (Table 2). All variables were adjusted for these differences in body weight in the statistical analysis (see Statistical methods). There was no significant difference in body weight between groups after either diet, or differences in the change of body weight between groups on either diet, over time.

**Table 2:** Effects of high and low sugars diets on anthropometrics and plasma lipids

	NAFLD (*n* = 11)		Controls (*n* = 14)	
	High sugars	Low sugars	High sugars	Low sugars
Body weight (kg)	89.8 ± 2.5	87.7 ± 2.4^4^	88.9 ± 2.8	86.7 ± 2.9^5^
BMI (kg/m^2^)	28.8 ± 0.4	28.2 ± 0.5	28.1 ± 0.6	27.4 ± 0.6
Liver fat^1^ (%)	24.2 ± 6.8	14.2 ± 3.2	3.6 ± 1.3	1.5 ± 0.3
Body fat^2^ (%)	27.3 ± 0.8	26.5 ± 0.9	24.8 ± 0.7	23.8 ± 0.9
Plasma TAG^3^ (mmol/l)	2.05 ± 0.24^6^	1.77 ± 0.22	1.33 ± 0.15	1.13 ± 0.08
Plasma cholesterol (mmol/l)	5.59 ± 0.33	5.24 ± 0.30	5.10 ± 0.25	4.82 ± 0.26
Plasma LDL-C (mmol/l)	3.40 ± 0.26	3.23 ± 0.28	3.27 ± 0.19	3.13 ± 0.21
Plasma HDL-C (mmol/l)	1.21 ± 0.09	1.15 ± 0.07	1.19 ± 0.07	1.16 ± 0.08
Plasma glucose (mmol/l)	5.35 ± 0.09	5.39 ± 0.09	5.08 ± 0.11	5.11 ± 0.08
Plasma insulin (mU/l)	21.2 ± 2.6	21.4 ± 1.0	17.9 ± 1.4	17.7 ± 2.4
HOMA2-IR	2.72 ± 0.33	2.76 ± 0.12	2.28 ± 0.17	2.26 ± 0.29

Values are arithmetic means ± SEMs unless stated otherwise. ^1^Measured by MRS on subgroup *n* = 17; ^2^measured by bio-electric impedance; ^3^geometric mean ± SEM. Significant difference between diets (within group): ^4^*P*<0.001; ^5^*P*<0.01. Significant difference between groups (within diet): ^6^*P*<0.02. All differences adjusted for body weight.

### Plasma lipids and lipoprotein kinetics

#### Summary of model interactions

There was an overall difference in the response to the two diets between the NAFLD and control groups, as evidenced by significant Group (NAFLD vs control) × diet (high vs low sugars) interactions for our primary outcome variables. These interactive variables included: (1) the plasma concentration and PR of large, TAG-rich VLDL_1_-TAG (*P* = 0.026, *P* = 0.015), which were higher in NAFLD compared with controls, but which increased in the controls in response to the high sugar diet; (2) the rate of VLDL_2_-TAG production (*P* = 0.04), which was higher in NAFLD than controls after the high sugar diet; (3) the rate of removal of plasma small, dense LDL_3_-apo B (*P* = 0.02), which was lower in NAFLD than controls after the low sugar diet; (4) plasma NEFA (*P* = 0.004) which was higher in NAFLD than controls after the high sugar diet and (5) the contribution of DNL to VLDL_1_TAG production (*P* = 0.02), which tended to be greater in controls after the high versus the low sugar diet, and higher in NAFLD relative to controls after the low sugar diet.

#### Post-hoc differences between groups

**Figure 1:**
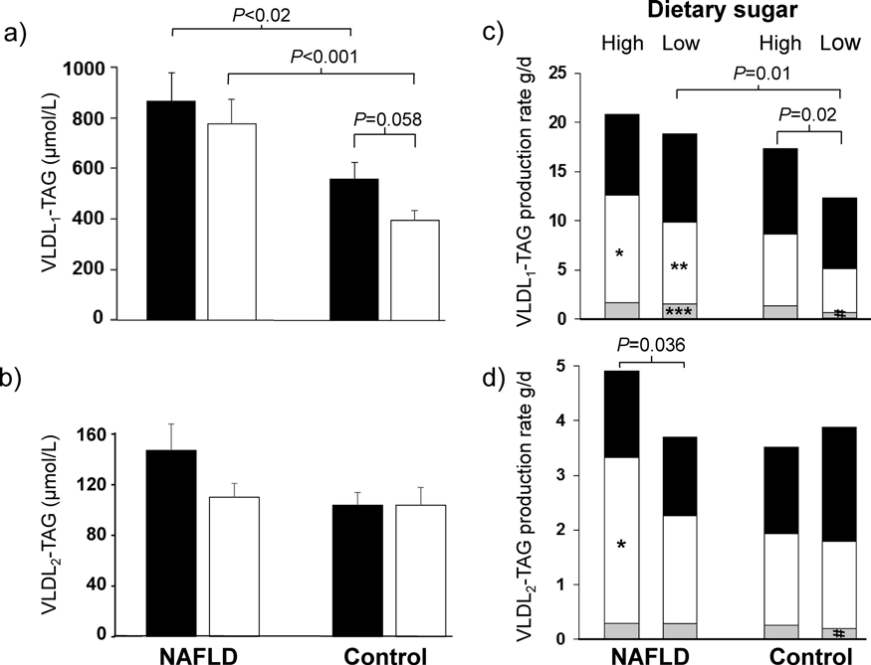
Effects of high and low sugar diets on the plasma concentration and source of fatty acids for the production rates of VLDL subclasses, in men with NAFLD and controls. Effects of high and low sugar diets (black and white bars, respectively) in men with NAFLD and low liver fat controls on the plasma concentrations of: (**a**) VLDL_1_-TAG and (**b**) VLDL_2_-TAG. Effects of high and low sugar diets on the contribution of fatty acids from systemic (black bars), splanchnic (white bars) and DNL (grey bars) to: (**c**) VLDL_1_-TAG production rate and (**d**) VLDL_2_-TAG production rate. Significance of weight-adjusted differences between groups and diets are as shown, and for differences between groups; *P<0.05; **P = 0.006; ***P = 0.003. **^#^***P* = 0.08 denotes trend for difference between diets in controls.

Men with NAFLD had higher plasma concentrations of total VLDL-TAG and VLDL_1_-TAG than controls, after the high (*P*<0.02 for both comparisons), and low (*P*<0.001, for both comparisons) sugar diets (Table 3, Figure 1a), and a higher VLDL_1_-TAG PR and lower VLDL_1_-TAG FCR than controls, after the low and high sugar diets, respectively (Figure 1c, and Table 4, *P* = 0.01 for both comparisons). Men with NAFLD also had a higher concentration of plasma small, dense LDL cholesterol (sdLDL), and lower FCR for small, dense LDL_3_-apo B than controls, after the low sugar diet (*P*<0.05 for both comparisons) (Tables 3 and 4).

**Table 3:** Effects of high and low sugars diets on plasma lipoprotein fraction concentrations

	NAFLD (*n* = 11)		Controls (*n* = 14)	
	High sugars	Low sugars	High sugars	Low sugars
Total VLDL-TAG (μmol/l)^1^	996 ±142^2^	872 ± 117^3^	651 ± 72	490 ± 51
VLDL_1_-TAG (μmol/l)	849 ± 109^2^	761 ± 97^3^	547 ± 67	386 ± 39
VLDL_2_-TAG (μmol/l	147 ± 21	110 ± 10	104 ± 11	104 ± 14
IDL-TAG (μmol/l)	61 ± 5	52 ± 5	54 ± 5	65 ± 11
VLDL-Chol (μmol/l)	509 ± 121^4^	381 ± 70^3^	290 ± 30	206 ± 23
VLDL_1_-Chol (μmol/l)	345 ± 76	283 ± 49	207 ± 26	127 ± 14
VLDL_2_-Chol (μmol/l)	163 ± 45	97 ± 13	82 ± 10	80 ± 13
IDL-chol (μmol/l)	167 ± 53	88 ± 13	88 ± 11	99 ± 16
VLDL_1_-apoB (mg/l)	15.6 ± 2.5	17.4 ± 3.0	15.1 ± 2.6	11.5 ± 1.9
VLDL_2_-apoB (mg/l)	12.5 ± 2.1	11.6 ± 1.5	13.1 ± 3.4	11.0 ± 2.7
IDL-apoB (mg/l)	21.9 ± 4.6	14.1 ± 1.8^5^	20.2 ± 5.0	20.9 ± 5.8
LDL-TAG (μmol/l)	1231 ± 165^2^	1088 ± 130^6^	843 ± 71	705 ± 57
LDL_2_-TAG (μmol/l)	99 ± 10	93 ± 13	75 ± 12	71 ± 8
LDL_3_-TAG (μmol/l)	79 ± 12	72 ± 8	60 ± 7	65 ± 6
LDL_2_-chol (μmol/l)	1019 ± 87	931 ± 106	781 ± 95	881 ± 82
LDL_3_-chol (μmol/l)	1222 ± 68	1252 ± 60	1141 ± 94	1172 ± 45
LDL_2_-apoB (mg/l)	306 ± 53	255 ± 40	258 ± 33	249 ± 32
LDL_3_-apoB (mg/l)	567 ± 92	574 ± 98	570 ± 48	459 ± 53
Small dense LDL (μmol/l)	1459 ± 210	1228 ± 175^4^	1043 ± 112	848 ± 78

^1^Sum of VLDL_1_ and VLDL_2_-TAG. Values are mean ± SEM. Significant difference between groups (within diet) ^2^*P*<0.02; ^3^*P*<0.001; ^4^*P*<0.05; ^6^*P*<0.005. Significant difference between diets (within group) ^5^*P*<0.05. All differences were adjusted for body weight.

**Table 4:** Effects of high and low sugars diets on lipoprotein kinetics and DNL

	NAFLD (*n* = 11)		Controls (*n* = 14)	
	High sugars	Low sugars	High sugars	Low sugars
VLDL_1_-TAG production rate (g/day)	20.9 ± 2.1	18.9 ± 2.1^2^	16.6 ± 1.4	12.4 ± 1.2^3^
VLDL_1_-TAG FCR (pools/day)^1^	9.0 ± 0.9^2^	9.5 ± 1.0	11.3 ± 0.7	11.9 ± 0.8
VLDL_2_-TAG production rate (g/day)	4.90 ± 0.59	3.70 ± 0.43^4^	3.63 ± 0.27	3.98 ± 0.43
VLDL_2_-TAG FCR (pools/day)	11.5 ± 1.1	12.2 ± 1.3	13.1 ± 1.0	14.3 ± 0.9
VLDL_1_-apoB production rate (mg/day)	481 ± 76	492 ± 58	546 ± 56	414 ± 54
VLDL_1_-apoB FCR (pools/day)	9.0 ± 1.0	10.8 ± 2.2	14.7 ± 2.7	13.4 ± 2.4
VLDL_2_-apoB production rate (mg/day)	546 ± 176	498 ± 164	720 ± 310	647 ± 212
VLDL_2_-apoB FCR (pools/day)	12.5 ± 2.8	12.8 ± 2.4	13.6 ± 1.9	14.9 ± 1.6
IDL-apoB production rate (mg/day)	609 ± 122	391 ± 69^5^	740 ± 159	737 ± 213
IDL-apoB FCR (pools/day)	9.5 ± 1.9	8.7 ± 0.9	12.2 ± 1.1	12.1 ± 1.2
LDL_2_-apoB production rate (mg/day)	1452 ± 277	858 ± 101	1075 ± 109	1176 ± 118
LDL_2_-apoB FCR (pools/day)	1.59 ± 0.25	1.35 ± 0.23	1.59 ± 0.24	1.74 ± 0.26
LDL_3_-apoB production rate (mg/day)	2069 ± 388	942 ± 278	1518 ± 237	1374 ± 273
LDL_3_-apoB FCR (pools/day)	1.01 ± 0.15	0.46 ± 0.09^6^	0.86 ± 0.12	1.06 ± 0.24
Contribution of DNL to:	1.66 ± 0.39	1.59 ± 0.34^3^	1.32 ± 0.43	0.56 ± 0.14^7^
VLDL_1_-TAG production (g/day)	1.66 ± 0.39	1.59 ± 0.34^7^	1.32 ± 0.43	0.56 ± 0.14
VLDL_2_-TAG production (g/day)	0.28 ± 0.05	0.29 ± 0.05	0.26 ± 0.05	0.19 ± 0.03

^1^Analysed for first period only, so between group comparisons (within diet) only were analysed (NAFLD; high sugar *n* = 7, low sugar *n* = 4. Controls; high sugar *n* = 7, low sugar *n* = 7). Values are mean ± SEM. Significant differences between groups (within diet) ^2^*P* = 0.01; ^6^*P*<0.05; ^7^*P* = 0.003. Significant differences between diets (within group) ^3^*P* = 0.02; ^4^*P* = 0.036; ^5^*P* = 0.06. All differences were adjusted for body weight. For the IDL and LDL_2_ kinetic data NAFLD (*n* = 9), and *n* = 8 for the LDL_3_ kinetic data due to insufficient data for the model fit.

#### Post-hoc differences between diets

Men with NAFLD had a higher PR of VLDL_2_-TAG (*P* = 0.036, Table 4, Figure 1d) after the high versus the low sugar diet. In contrast, controls had a significantly higher PR of VLDL_1_-TAG (*P* = 0.02), and trend towards a higher plasma concentration of VLDL_1_-TAG (*P* = 0.058), after the high versus low sugar diet (Figure 1 c,a). Men with NAFLD had a higher plasma concentration of IDL-apo B (*P* = 0.025, Table 3), IDL-apo B pool size (*P* = 0.025, data not shown), and trend for a higher IDL-apo B PR (*P* = 0.06, Table 4), after the high versus the low sugar diet.

### Sources of fatty acids for VLDL production

#### Post-hoc differences between groups

Men with NAFLD had a greater contribution of fatty acids from splanchnic fat for the production of VLDL_1_ and VLDL_2_-TAG relative to controls, after the high sugars diet (Figure 1c,d, *P*<0.05 for both comparisons). This group also expressed a greater contribution of fatty acids from splanchnc fat, and DNL for the production of VLDL_1_-TAG after the low sugars diet (Figure 1c, *P* = 0.006, *P* = 0.003, respectively), and a markedly higher plasma concentration of NEFA after the high sugars diet, relative to controls (*P* = 0.0007, Table 5).

**Table 5:** Effects of high and low sugars diets on palmitate kinetics, post-heparin lipase activities and plasma apoproteins

	NAFLD (*n* = 11)		Controls (n = 14)	
	High sugars	Low sugars	High sugars	Low sugars
Plasma NEFA (μmol/l)	658 ± 30^1^	548 ± 44	438 ± 31	526 ± 42^2^
Plasma Palmitate (μmol/l)	220 ± 39	238 ± 25	214 ± 25	218 ± 28
Palmitate production rate (μmol/min)	169 ± 11	147 ± 12^3^	168 ± 15	168 ± 17
Palmitate MCR (ml/min)	863 ± 74	647 ± 56^4^	863 ± 110	850 ± 101
Post heparin LPL (pmol/ml/min)	1.33 ± 0.31	1.30 ± 0.21	1.36 ± 0.19	1.97 ± 0.32
Post heparin HL (pmol/ml/min)	2.13 ± 0.48^2^	1.43 ± 0.38	1.01 ± 0.17	0.90 ± 0.18
Plasma apoE (mg/l)	33.3 ± 3.7	30.2 ± 2.7	29.1 ± 1.4	27.7 ± 1.4
Plasma apo C-III (mg/l)	112.2 ± 9.8^2^	103.8 ± 9.1^5^	86.0 ± 7.5	73.5 ± 5.4
Plasma apo C-II (mg/l)	82.7 ± 9.2	77.0 ± 8.0^2^	61.7 ± 6.1	56.9 ± 5.6

Values are mean ± SEM. Significant difference between groups (within diet): ^1^*P*<0.001; ^2^*P*<0.05; ^5^*P*<0.01 Significant difference between diets (within group); ^3^*P*<0.05; ^4^*P*<0.01. All differences were adjusted for body weight.

#### Post-hoc differences between diets

There were no significant effects of diet on the source of fatty acids for VLDL production, other than a trend for a greater contribution from DNL to the production of VLDL_1_ and VLDL_2_-TAG in controls, after the high versus the low sugars diet (*P* = 0.08 for both VLDL subclasses). The production and metabolic clearance rates of palmitate were higher in men with NAFLD (*P* = 0.025, *P* = 0.006, respectively), after the high versus low sugars diet (Table 5).

### Plasma apoproteins and post-heparin lipase activities

#### Post-hoc differences between groups

Men with NAFLD had a higher plasma apoprotein C-III than controls, after the high and low sugars diets (*P* = 0.042, *P* = 0.002, respectively), and a higher plasma apoprotein C-II than controls after the low sugars diet (*P* = 0.033). The activity of HL was higher in men with NAFLD versus controls after the high sugars diet (*P*<0.05) (Table 5).

#### Liver fat, intra-abdominal and subcutaneous adipose tissue (subgroup n = 17, post-diet)

Liver fat was higher after the high sugars diet in men with NAFLD and controls, relative to the low sugars diet (*P* = 0.01 for both comparisons, Table 2). However, the significance of these differences was not maintained after adjustment for body weight. There were no differences in the masses of visceral and subcutaneous adipose tissue between groups after each diet (Supplementary Table S3). There were also no associations between post-dietary liver fat, body weight, visceral fat, plasma TAG, or changes in these variables.

## Discussion

**Figure 2:**
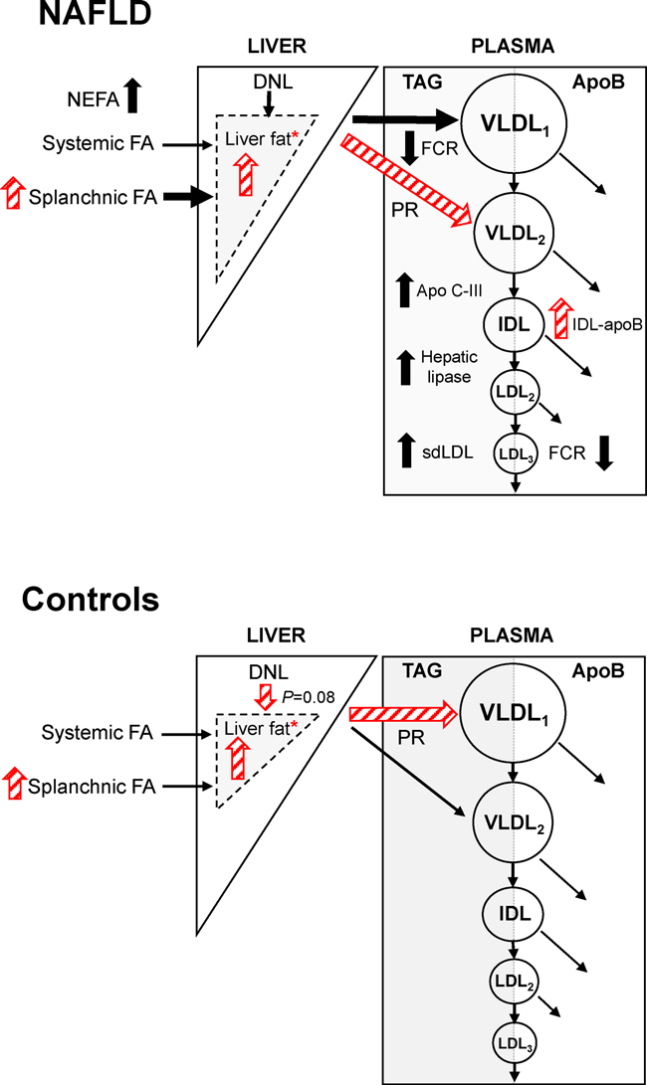
Summary schematic of the relative effects of the high and low sugar diets on the lipoprotein metabolism of men with NAFLD and low liver fat controls The relative effects of the high and low sugar diets on lipoprotein metabolism are shown as red hatched arrows. The thickness of black arrows represents the magnitude of pathway in men with NAFLD relative to controls (PR, production rate; FCR, fractional catabolic rate). *Significance of increases in liver fat in both NAFLD and controls, after the high sugar diet relative to the low sugar diet, were not maintained after adjustment for body weight.

The present study provides new evidence that liver fat can influence the weight-adjusted partitioning of hepatic TAG into different plasma VLDL subclasses, in response to a high intake of sugars that is common to the UK diet [13]. Men with NAFLD were distinct from controls in having a higher plasma and PR of large, TAG-rich VLDL_1_, after both diets. This finding is consistent with the previous observation that VLDL_1_ overproduction is driven by increased liver fat [20]. In the present study, this effect originated, in part, from a greater contribution of fatty acids from splanchnic fat (hepatic TAG storage pools, visceral fat, and to a lesser extent DNL in the liver). A highly original finding in the present study, was that these metabolic characteristics in men with NAFLD were shown to develop in response to the high sugars diet in low liver fat controls. In contrast, the high sugars diet up-regulated the production of VLDL_2_ in NAFLD relative to controls, a difference that was also ascribed to a greater contribution of splanchnic fatty acids for the production of this smaller VLDL subclass (Figure 2).

Large TAG-rich VLDL_1_ has been associated with increased liver fat and dyslipidaemia in the metabolic syndrome [20,21], but there is no previous evidence to link its plasma concentration or kinetics directly with a high intake of sugars in humans. There have also been no studies to date on the effect of dietary free sugars on VLDL kinetics in NAFLD. In healthy subjects, the PR of VLDL-TAG has been shown to be higher after a 6-day hyper-energetic diet enriched with fructose as a liquid supplement (25% total energy) versus a 6-day, low-fructose diet [22]. VLDL-TAG PR was also higher after a 2-week high carbohydrate, low fat diet, compared with a 2-week iso-energetic, low carbohydrate, high fat diet in healthy subjects [23]. In the present study, the PR and plasma concentration of large, TAG-rich VLDL_1_ were higher in the low liver fat controls on the high sugars diet compared with the low sugars diet. Moreover, the difference in PR of large TAG-rich VLDL_1_ between groups was removed on the high sugars diet, as the values in controls approached that of men with NAFLD, possibly because the controls also gained liver fat. In contrast, the PR of smaller VLDL_2_-TAG was significantly higher in NAFLD after the high relative to the low sugar diet. Since VLDL_2_ is known to be the main precursor of IDL and LDL [24], this finding is consistent with an increase in IDL apoB PR and the pool size of IDL apoB and plasma concentration of apoB in IDL, and small dense low density lipoprotein (sdLDL), both of which are components of an ALP [25]. Interestingly, there was no evidence in our study of any group or dietary effects on the production and secretion of new VLDL particles, as indicated by a lack of significant effects on plasma VLDL apoB or changes in the kinetics of VLDL-apoB.

Men with NAFLD had a higher DNL relative to controls after the low sugars diet, in accord with previous reports of increased contribution of DNL to hepatic fat and dyslipidaemia in men with NAFLD [8,26]. However, this finding was only significant on the low sugars diet, possibly because the contribution of DNL to both VLDL_1_ and VLDL_2_-TAG increased to a greater extent in controls than in men with NAFLD after the high sugars diet. DNL made relatively minor contributions (between 4 and 8%) to VLDL_1_ and VLDL_2_-TAG production in both groups, after both diets, as reported previously in healthy subjects [27]. DNL has been shown to contribute approximately 12% of palmitate to VLDL-TAG in a previous study in NAFLD, when measured over a comparable time period to the present study [8]. In a previous study, an 8 weeks diet with fructose-sweetened beverages, providing 25% of total energy, increased DNL, whereas glucose-sweetened beverages had no effect in the healthy overweight participants [28]. Similarly, a 6-day high-fructose diet (25% total energy) was shown to increase DNL from 1.6 to 9.4% in VLDL-palmitate in healthy, normal weight men [29].

In the present study, there was no significant difference in the systemic contribution of fatty acids to VLDL_1_-TAG or VLDL_2_-TAG production between the diets in either group. This is perhaps surprising, given the marked increase in plasma NEFA and higher production and clearance rates of palmitate after the high sugars diet in the NAFLD group, which might be expected to increase the delivery of NEFA to the liver. There was, however, a greater contribution of splanchnic fat to VLDL_1_-TAG and VLDL_2_-TAG production in NAFLD relative to controls, which might help to explain how liver fat influences the differential partitioning of hepatic TAG in these groups in response to dietary sugars.

Splanchnic fat includes hepatic TAG storage pools and visceral adipose tissue, the NEFA from which drains directly into the liver via the portal vein. Hepatic TAG storage pools will expand in the fed, postprandial state, with an estimated 22% of dietary TAG being taken-up by the liver in chylomicron remnants [30], some of which will be stored and contribute to VLDL synthesis in the post-absorptive state [31].

The flux of NEFA from visceral adipose tissue has been estimated to be 20% of total NEFA delivery to the liver in obese subjects, but only 5% in lean subjects [32,33] based on a model partially validated in dogs [34]. Visceral adipose NEFA flux was also shown to correlate with visceral fat measured by computer tomography [32].

Since the men in our study were generally overweight, but not obese, visceral adipose tissue is likely to have made a small contribution (5–20%) towards the delivery of total NEFA to the liver [32,33]. However, since visceral fat was not different between groups and unaffected by the diets in the present study, this suggests that the relatively greater contribution of splanchnic-derived NEFAs to VLDL_1_ and VLDL_2_-TAG production on the high sugars diet in NAFLD relative to controls, came from hepatic TAG storage pools. This possibility introduces the established effect of dietary sugars in augmenting post-prandial lipaemia [35], and highlights the importance of postprandial TAG as a potential source of lipid for the accumulation of liver fat [36]. While postprandial responses were not measured in our study, the high sugar diet increased VLDL_1_ in controls, and serum apo C-III in NAFLD, an apoprotein with roles in the assembly of VLDL_1_ in the liver and inhibition of LPL [37]. These effects are consistent with dietary free sugars impairing the clearance of plasma TAG in the postprandial phase [35,36].

The intake of sugars on the low sugars diet was close to the current recommendation for the intake of free sugars, of no more than 5% total energy (NMES 6 ± 2% total energy or 586 kJ (140 kcal)/day) [38,39]. In contrast, the intake of sugars on the high sugars diet (NMES 26 ± 7% total energy) was five-fold greater than this recommendation (2721 kJ (650 kcal)/day), but still within the upper 2.5th percentile of intake in a typical UK diet [13]. Although we cannot exclude the possibility that the small differences in the intake of dietary fat between the iso-energetic diets contributed to the metabolic effects (5% and 8% energy in NAFLD and controls, respectively), the overall, weight-adjusted response of outcome variables is consistent with the marked differences in intake of dietary sugars between the two diets (19% and 20% energy, in NAFLD and controls, respectively).

It is well documented that hyper-energetic diets, high in sugars, increase liver fat in healthy men [40], but there is less evidence that iso-energetic diets, high in sugars, exert the same effect. A weight-maintaining high fructose diet (25% total energy) has been reported to increase liver fat by 137% in healthy men [41]. Similarly, an iso-energetic diet containing sucrose-sweetened regular cola increased liver fat by 132% in overweight subjects [42]. In the present study, the high sugars diet increased liver fat to a relatively greater extent in subgroups of men with NAFLD, compared with controls. While this might suggest greater sensitivity to dietary sugars in NAFLD, the statistical significance of this difference in liver fat was lost after adjustment for the small gain in body weight. This finding reaffirms that liver fat is very sensitive to increased body weight in response to dietary sugars [43].

Strengths of our study include the dietary exchange, which achieved its targets for sugar intake in a free-living setting, and stable isotope trace-labelling methodology to simultaneously track the metabolism of plasma lipoproteins, fatty acids and DNL. Limitations of our study include its sample size, and the dependence of our main outcome measures on the assumptions inherent in mathematical modelling. In addition, results derived from the infusion of stable isotope labelled palmitate are dependent on the validity of assumptions regarding fatty acid fluxes to the liver. While we adjusted all data for the small and consistent changes in body weight in response to differences in energy intake between diets, we cannot exclude the possibility of acute metabolic effects arising from these differences. Nevertheless, the overall pattern of metabolic responses to the diets, and significance of weight-adjusted differences in our outcome variables, including VLDL_1_-TAG PR, on which the sample size was originally powered, provide confidence that these data are robust. It also suggests that the effects of a high and low intake of sugars on lipoprotein metabolism were independent of the relationship between changes in body weight and liver fat.

The present study provides new evidence that liver fat influences the effects of dietary free sugars in partitioning plasma TAG into different VLDL subclasses. This finding has major implications for the potential mechanism by which dietary free sugars could contribute to the development of NAFLD, and dyslipidaemia.

## Clinical perspectives

- A high intake of dietary sugars consumed in foods and sugar sweetened beverages, has been implicated in the development of fatty liver disease, possibly through adverse effects on lipid metabolism. The present study was undertaken to determine if liver fat influences the plasma lipid and lipoprotein response to sugars, and the mechanism by which sugars contribute to the accumulation of liver fat.
- High and low sugar diets produced differential effects on the metabolism of plasma VLDL subclasses in men with raised liver fat (NAFLD) and low liver fat controls. A high intake of sugars produced changes in the lipoprotein metabolism of controls that were characteristic of men with NAFLD.
- These findings indicate that the accumulation of liver fat can influence the plasma lipid and lipoprotein response to dietary sugars, and provide new evidence for a mechanism to explain how sugars may contribute to NAFLD and dyslipidaemia.

## Supporting information

**Figure S1:** Protocol for metabolic study

**Figure S2:** Schematic of model used to describe TTR_s_ of VLDL_1_ and VLDL_2_-TAG.

**Figure S3:** Schematic description of the model used to describe TTRs of VLDL_1_ VLDL_2_, IDL, LDL_2_ and LDL_3_-apoB.

**Table S1:** Intake of energy and macronutrients

**Table S2:** Body fat distribution measured by MRS

## Abbreviations

ALP: atherogenic lipoprotein phenotype
apo: apolipoprotein
DNL: de novo lipogenesis
FCR: fractional catabolic rate
GCMS: gas chromatography mass spectrometry
HL: hepatic lipase
IDL: Intermediate density lipoprotein
IHCL: intra-hepatocellular lipid
LPL: lipoprotein lipase
MRI: magnetic resonance imaging
MRS: magnetic resonance spectroscopy
NAFLD: non-alcoholic fatty liver disease
NDNS: National Diet & Nutrition Survey
NMES: non-milk extrinsic sugars
PR: production rate
sdLDL: small dense low density lipoprotein
TAG: triacylglycerol
TTR: tracer/tracee ratio
VLDL: very low density lipoprotein

## References

[1] Hashimoto E., Tokushige K. and Ludwig J. (2015) Diagnosis and classification of non-alcoholic fatty liver disease and non-alcoholic steatohepatitis: current concepts and remaining challenges. Hepatol. Res. 45, 20–28 24661406 10.1111/hepr.12333

[2] Stanhope K.L., Schwarz J.M. and Havel P.J. (2013) Adverse metabolic effects of dietary fructose: results from the recent epidemiological, clinical, and mechanistic studies. Curr. Opin. Lipidol. 24, 198–206 23594708 10.1097/MOL.0b013e3283613bca PMC4251462

[3] Te Morenga L.A., Howatson A.J., Jones R.M. and Mann J. (2014) Dietary sugars and cardiometabolic risk: systematic review and meta-analyses of randomized controlled trials of the effects on blood pressure and lipids. Am. J. Clin. Nutr. 100, 65–79 24808490 10.3945/ajcn.113.081521

[4] Lonardo A., Sookoian S., Pirola C.J. and Targher G. (2016) Non-alcoholic fatty liver disease and risk of cardiovascular disease. Metabolism 65, 1136–1150 26477269 10.1016/j.metabol.2015.09.017

[5] Griffin B.A., Freeman D.J., Tait G.W., Thomson J, Caslake M.J., Packard C.J. (1994) Role of plasma triglyceride in the regulation of plasma low density lipoprotein (LDL) subfractions: relative contribution of small, dense LDL to coronary heart disease risk. Atherosclerosis 106, 241–253 8060384 10.1016/0021-9150(94)90129-5

[6] Taskinen M.R., Adiels M., Westerbacka J., Söderlund S., Kahri J., Lundbom N. (2011) Dual metabolic defects are required to produce hypertriglyceridemia in obese subjects. Arterioscler. Thromb. Vasc. Biol. 31, 2144–2150 21778423 10.1161/ATVBAHA.111.224808

[7] Watts G.F., Mandalia S., Brunt J.N., Slavin B.M., Coltart D.J. and Lewis B. (1993) Independent associations between plasma lipoprotein subfraction levels and the course of coronary artery disease in the St. Thomas' Atherosclerosis Regression Study (STARS). Metabolism 42, 1461–1467 8231842 10.1016/0026-0495(93)90199-x

[8] Donnelly K.L., Smith C.I., Schwarzenberg S.J., Jessurun J., Boldt M.D. and Parks E.J. (2005) Sources of fatty acids stored in liver and secreted via lipoproteins in patients with nonalcoholic fatty liver disease. J. Clin. Invest. 115, 1343–1351 15864352 10.1172/JCI23621 PMC1087172

[9] Stanhope K.L. (2016) Sugar consumption, metabolic disease and obesity: the state of the controversy. Crit. Rev. Clin. Lab. Sci. 53, 52–67 26376619 10.3109/10408363.2015.1084990 PMC4822166

[10] Schwarz J.M., Neese R.A., Turner S., Dare D. and Hellerstein M.K. (1995) Short-term alterations in carbohydrate energy intake in humans. Striking effects on hepatic glucose production, de novo lipogenesis, lipolysis, and whole-body fuel selection. J. Clin. Invest. 96, 2735–2743 8675642 10.1172/JCI118342 PMC185982

[11] Jebb S.A., Lovegrove J.A., Griffin B.A., Frost G.S., Moore C.S., Chatfield M.D. (2010) Effect of changing the amount and type of fat and carbohydrate on insulin sensitivity and cardiovascular risk: the RISCK (Reading, Imperial, Surrey, Cambridge, and Kings) trial. Am. J. Clin. Nutr. 92, 748–758 20739418 10.3945/ajcn.2009.29096 PMC3594881

[12] Szczepaniak L.S., Nurenberg P., Leonard D., Browning J.D., Reingold J.S., Grundy S. (2005) Magnetic resonance spectroscopy to measure hepatic triglyceride content: prevalence of NAFLD in the general population. Am. J. Physiol. Endocrinol. Metab. 288, E462–E468 15339742 10.1152/ajpendo.00064.2004

[13] National Diet and Nutrition Survey (2014) Results from Year 1–4 (combined) of the Rolling Programme (2008/2009–2011/2012), Public Health England

[14] Department of Health (1989) Dietary Sugars and Human Disease. Committee on Medical Aspects of Food Policy. Report on Health and Social Subjects No. 37, HMSO, London

[15] Kelly S.A., Summerbell C., Rugg-Gunn A.J., Adamson A., Fletcher E. and Moynihan P.J. (2005) Comparison of methods to estimate non-milk extrinsic sugars and their application to sugars in the diet of young adolescents. Br. J. Nutr. 94, 114–124 16115340 10.1079/bjn20051448

[16] Sarac I., Backhouse K., Shojaee-Moradie F., Stolinski M., Robertson M.D., Bell J.D. (2012) Gender differences in VLDL1 and VLDL2 triglyceride kinetics and fatty acid kinetics in obese postmenopausal women and obese men. J. Clin. Endocrinol. Metab. 97, 2475–2481 22508714 10.1210/jc.2011-3248

[17] Brackenridge A.L., Jackson N., Jefferson W., Stolinski M., Shojaee-Moradie F, Hovorka R. (2009) Effects of rosiglitazone and pioglitazone on lipoprotein metabolism in patients with Type 2 diabetes and normal lipids. Diabet. Med. 26, 532–539 19646194 10.1111/j.1464-5491.2009.02729.x

[18] McGee K.C., Shahmanesh M., Boothby M., Nightingale P., Gathercole L.L., Tripathi G., Evidence for a shift to anaerobic metabolism in adipose tissue in efavirenz-containing regimens for HIV with different nucleoside backbones. Antivir. Ther. 17, 495–507 22300946 10.3851/IMP2017

[19] Thomas E.L., Hamilton G., Patel N., O'Dwyer R., Doré C.J., Goldin R.D. (2005) Hepatic triglyceride content and its relation to body adiposity: a magnetic resonance imaging and proton magnetic resonance spectroscopy study. Gut 54, 122–127 15591516 10.1136/gut.2003.036566 PMC1774370

[20] Adiels M., Taskinen M.R., Packard C.J., Caslake M.J., Soro-Paavonen A., Westerbacka J. (2006) Overproduction of large VLDL particles is driven by increased liver fat content in man. Diabetologia 49, 755–765 16463046 10.1007/s00125-005-0125-z

[21] Adiels M., Olofsson S.O., Taskinen M.R. and Borén J. (2008) Overproduction of very low-density lipoproteins is the hallmark of the dyslipidemia in the metabolic syndrome. Arterioscler. Thromb. Vasc. Biol. 28, 1225–1236 18565848 10.1161/ATVBAHA.107.160192

[22] Theytaz F., Noguchi Y., Egli L., Campos V., Buehler T., Hodson L. (2012) Effects of supplementation with essential amino acids on intrahepatic lipid concentrations during fructose overfeeding in humans. Am. J. Clin. Nutr. 96, 1008–1016 23034968 10.3945/ajcn.112.035139

[23] Mittendorfer B. and Sidossis L.S. (2001) Mechanism for the increase in plasma triacylglycerol concentrations after consumption of short-term, high-carbohydrate diets. Am. J. Clin. Nutr. 73, 892–899 11333842 10.1093/ajcn/73.5.892

[24] Gaw A., Packard C.J., Lindsay G.M., Griffin B.A., Caslake M.J., Lorimer A.R. (1995) Overproduction of small very low density lipoproteins (Sf 20-60) in moderate hypercholesterolemia: relationships between apolipoprotein B kinetics and plasma lipoproteins. J. Lipid Res. 36, 158–171 7706941

[25] Griffin B.A. and Zampelas A. (1995) Influence of dietary fatty acids on the atherogenic lipoprotein phenotype. Nutr. Res. Rev. 8, 1–26 19094277 10.1079/NRR19950004

[26] Lambert J.E., Ramos-Roman M.A., Browning J.D. and Parks E.J. (2014) Increased de novo lipogenesis is a distinct characteristic of individuals with nonalcoholic fatty liver disease. Gastroenterology 146, 726–735 24316260 10.1053/j.gastro.2013.11.049 PMC6276362

[27] Timlin M.T. and Parks E.J. (2005) Temporal pattern of de novo lipogenesis in the postprandial state in healthy men. Am. J. Clin. Nutr. 81, 35–42 15640457 10.1093/ajcn/81.1.35

[28] Stanhope K.L., Schwarz J.M., Keim N.L., Griffen S.C., Bremer A.A., Graham J.L. (2009) Consuming fructose-sweetened, not glucose-sweetened, beverages increases visceral adiposity and lipids and decreases insulin sensitivity in overweight/obese humans. J. Clin. Invest. 119, 1322–1334 19381015 10.1172/JCI37385 PMC2673878

[29] Faeh D., Minehira K., Schwarz J.M., Periasamy R., Park S. and Tappy L. (2005) Effect of fructose overfeeding and fish oil administration on hepatic de novo lipogenesis and insulin sensitivity in healthy men. Diabetes 54, 1907–1913 15983189 10.2337/diabetes.54.7.1907

[30] Bergman E.N., Havel R.J., Wolfe B.M. and Bohmer T. (1971) Quantitative studies of the metabolism of chylomicron triglycerides and cholesterol by liver and extrahepatic tissues of sheep and dogs. J. Clin. Invest. 50, 1831–1839 5564390 10.1172/JCI106674 PMC292108

[31] Parks E.J., Krauss R.M., Christiansen M.P., Neese R.A. and Hellerstein M.K. (1999) Effects of a low-fat, high-carbohydrate diet on VLDL-triglyceride assembly, production, and clearance. J. Clin. Invest. 104, 1087–1096 10525047 10.1172/JCI6572 PMC408572

[32] Nielsen S., Guo Z., Johnson C.M., Hensrud D.D. and Jensen M.D. (2004) Splanchnic lipolysis in human obesity. J. Clin. Invest. 113, 1582–1588 15173884 10.1172/JCI21047 PMC419492

[33] Klein S. (2004) The case of visceral fat: argument for the defense. J. Clin. Invest. 113, 1530–1532 15173878 10.1172/JCI22028 PMC419497

[34] Jensen M.D, Cardin S., Edgerton D. and Cherrington A. (2003) Splanchnic free fatty acid kinetics. Am. J. Physiol. Endocrinol. Metab. 284, E1140–E1148 12736157 10.1152/ajpendo.00268.2002

[35] Abraha A., Humphreys S.M., Clark M.L., Matthews D.R. and Frayn K.N. (1998) Acute effect of fructose on postprandial lipaemia in diabetic and non-diabetic subjects. Br. J. Nutr. 80, 169–175 9828758

[36] Griffin B.A. (2015) Relevance of liver fat to the impact of dietary extrinsic sugars on lipid metabolism. Proc. Nutr. Soc. 74, 208–214 25992705 10.1017/S0029665115002050

[37] Yao Z. (2012) Human apolipoprotein C-III – a new intrahepatic protein factor promoting assembly and secretion of very low density lipoproteins. Cardiovasc. Hematol. Disord. Drug Targets 12, 133–140 23030451 10.2174/1871529x11202020133

[38] World Health Organisation's Draft Guidelines on sugar intake for adults and children (2014) https://www.who.int/nutrition/sugars_public_consultation/en/ (accessed 2 August 2015)

[39] Scientific Advisory Committee on Nutrition: Draft report on carbohydrates and health (2014) https://www.gov.uk/government/uploads/system/uploads/attachment_data/file/445503/SACN_Carbohydrates_and_Health.pdf (accessed 5 August 2015)

[40] Chung M., Ma J., Patel K., Berger S., Lau J. and Lichtenstein A.H. (2014) Fructose, high-fructose corn syrup, sucrose, and non-alcoholic fatty liver disease or indexes of liver health: a systematic review and meta-analysis. Am. J. Clin. Nutr. 100, 833–849 25099546 10.3945/ajcn.114.086314 PMC4135494

[41] Schwarz J.M., Noworolski S.M., Wen M.J., Dyachenko A., Prior J.L., Weinberg M.E. (2015) Effect of a high-fructose weight-maintaining diet on lipogenesis and liver fat. J. Clin. Endocrinol. Metab. 100, 2434–2442 25825943 10.1210/jc.2014-3678 PMC4454806

[42] Maersk M., Belza A., Stødkilde-Jørgensen H., Ringgaard S., Chabanova E., Thomsen H. (2012) Sucrose-sweetened beverages increase fat storage in the liver, muscle, and visceral fat depot: a 6-mo randomized intervention study. Am. J. Clin. Nutr. 95, 283–289 22205311 10.3945/ajcn.111.022533

[43] Sevastianova K., Santos A., Kotronen A., Hakkarainen A., Makkonen J., Silander K. (2012) Effect of short-term carbohydrate overfeeding and long-term weight loss on liver fat in overweight humans. Am. J. Clin. Nutr. 96, 727–734 22952180 10.3945/ajcn.112.038695

