# Supplementary material for: Impact of liver fat on the differential partitioning of hepatic triacylglycerol into VLDL subclasses on high and low sugar diets
Source: Clin Sci (Lond). 2017 Oct 17;131(21):2561–73. doi: 10.1042/CS20171208 (PMC6365592; doi:10.1042/CS20171208)

Supplementary Material

Impact of liver fat on the differential partitioning of hepatic triacylglycerol into very low density lipoprotein subclasses in response to high and low sugar diets

Authors: A. Margot Umpleby[1], Fariba Shojaee-Moradie[1], Barbara Fielding[1], Xuefei Li[1], Andrea Marino[1], Najlaa Alsini[1], Cheryl Isherwood[1], Nicola Jackson[1], Aryati Ahmad[1,2], Michael Stolinski[1], Julie A. Lovegrove[3], Sigurd Johnsen[1], A Mendis[1], John Wright[1], Malgorzata E Wilinska[4], Roman Hovorka[4], Jimmy D Bell[5,6], E Louise Thomas[5,6], Gary S Frost[7], Bruce A. Griffin[1]

Table of Contents (page numbers):

Dietary exchange model (pp. 2)

Figure: Protocol for metabolic study (pp. 2)

Data anaysis (pp. 3)

Kinetic modelling (pp. 4-6)

References (pp. 7)

Supplementary Table 1. Intake of energy and macronutrients (pp. 8)

Supplementary Table 2. Body fat distribution measured by MRS (pp.9)

Dietary exchange model

The sugar content of the two diets was achieved by a dietary exchange of sugar for starch using foods that were either high or low in total sugars (≥ 40% or ≤ 10% of total carbohydrate (CHO), respectively). Foods with intermediate sugar content were excluded from the dietary exchange model, the aim of which was to replace two thirds of the habitual CHO intake with study foods (approximately 180 g/day) without changing other dietary components. Participants were required to exchange 6 portions of their habitual CHO per day (a portion representing 30g CHO) with either the high or low sugar foods, depending on their allocated diet. A number of different foods and drinks (containing either high sugar/low starch or low sugar/high starch) were supplied to the participants, which allowed dietary flexibility and aided compliance. The intervention diets were designed to be matched for total carbohydrate, protein and fat content and iso-energetic. Five home visits were made every 2 weeks to supply study foods, measure body weight, and to assess daily food and drink portion sheets to help maintain dietary compliance, and to maintain body weight to within ± 0.5 kg.

Figure 1: Protocol for metabolic study

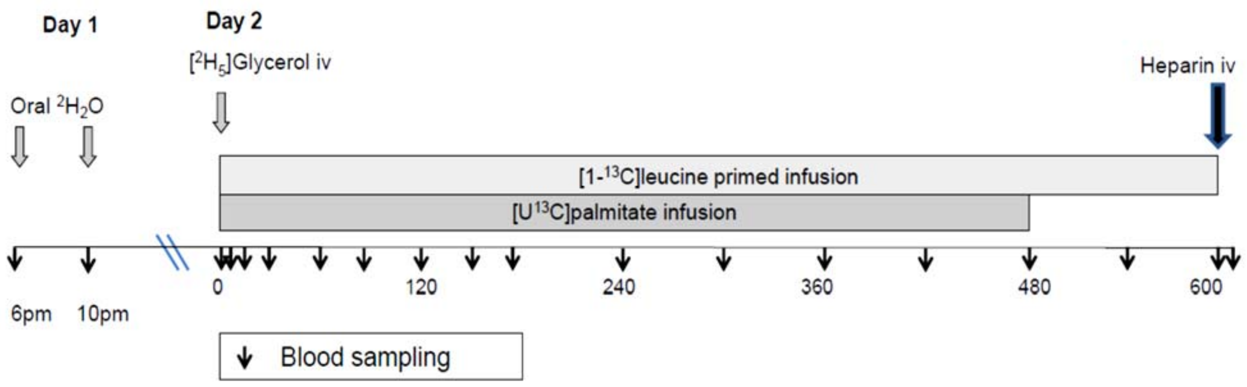

Data analysis (Sources of fatty acids for VLDL production)

Palmitate production rate (PR) was calculated as: ***Palmitate PR (μmol/min) = Infusion rate of palmitate tracer (μmol/min) /TTR_{SS}.*** Where TTR = m/z 286/270 at time t minus m/z 286/270 at t=0 min, TTR_{SS} = mean TTR $_{(t=420\text{-}480\ min)}$ and SS= steady state.

The contribution of circulating palmitate (systemic contribution) to $VLDL_1$-TAG PR was calculated as: ***Systemic contribution of fatty acids to $VLDL_1$-TAG PR (g/d) =$VLDL_1$-TAG PR (g/d) x (($VLDL_1$ TAG palmitate TTR_{SS} / plasma palmitate TTR_{SS}).*** This will include a contribution from visceral fat, since some labelled palmitate will be taken-up by this fat store and released into the portal vein.

The percent contribution of hepatic DNL-derived palmitate to $VLDL_1$ and $VLDL_2$-TAG PR was calculated from the deuterium enrichment in the palmitate of $VLDL_1$ and $VLDL_2$-TAG and in plasma water as previously described [1]. The calculation assumes that in all VLDL-TAG fatty acids derived from DNL, the enrichment in TG-palmitate (Maximum palmitate TTR) will be ***Maximum palmitate TTR = ^2H_2O TTR x N,*** where ^2H_2O TTR is the enrichment of the plasma water, and N is the maximum number of deuterium atoms, that can be incorporated into a molecule of palmitate. In the present study N was 21, based on previous observations [1]. The percentage of palmitate derived from DNL in VLDL-TAG was calculated as: ***% hepatic DNL in VLDL-TAG palmitate = (VLDL-TG palmitate TTR / maximum palmitate TTR) / 100*** where TTR is m/z 271/270 at time 12 hour minus 271/270 at time 0 minutes. For details of the time course of deuterium incorporation into VLDL-TAG palmitate see Diraison *et al.* (1997) [2].

The contribution of DNL to $VLDL_1$-TAG PR was estimated as: ***DNL contribution to $VLDL_1$-TAG PR (g/d) = % hepatic DNL x $VLDL_1$-TAG PR (g/d) x 100.***

The splanchnic fat contribution was assumed to be all other sources of fatty acids and was calculated as: ***Splanchnic fat contribution of fatty acids to $VLDL_1$-TAG PR (g/d) = $VLDL_1$-TAG PR (g/d) - (DNL (g/d) + Systemic (g/d)).***

In these calculations it was assumed that palmitate is a representative of NEFAs. $VLDL_2$-TAG PR was substituted for $VLDL_1$-TAG PR in the above equations to calculate the contribution of different fatty acid sources to $VLDL_2$-TAG PR.

Kinetic modelling of $VLDL_1$ and $VLDL_2$-TAG

$VLDL_1$-TAG and $VLDL_2$-TAG FCR were calculated using a compartment model of $VLDL_1$-TG and $VLDL_2$-TG kinetics using SAAM II software. The model represents the kinetics of the tracer-to-tracee ratio (TTR) profiles which change as labelled glycerol is removed from plasma and incorporated into the TAG fractions. Plasma glycerol kinetics was described by a sum of three exponentials representing a three compartment model. A five-compartment chain described a time delay due to synthesis and secretion of $VLDL_1$ and $VLDL_2$-TAG. The model is schematically depicted in Fig. 2.

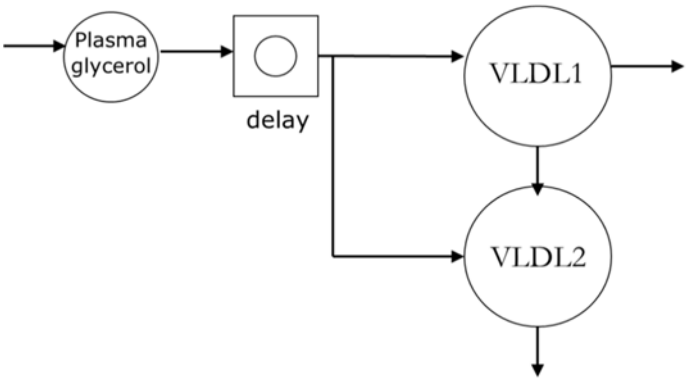

Figure 2. Schematic of model used to describe TTRs of $VLDL_1$ and $VLDL_2$-TAG.

The model assumes steady state of native (unlabelled) glycerol throughout the experimental period, i.e. a constant appearance, disappearance, and incorporation of native glycerol into the TAG fractions. The incorporation of glycerol into VLDL by the liver is subject to a delay. The model included a compartment for $VLDL_1$-TAG and a compartment for $VLDL_2$-TAG with an input into both compartments from the glycerol precursor pool, a loss from each compartment and a transfer from the $VLDL_1$-TAG compartment to the $VLDL_2$-TAG compartment. $VLDL_1$-TAG and $VLDL_2$-TAG production rates were calculated as the product of $VLDL_1$-TAG and $VLDL_2$-TAG FCR and their respective TAG pools. $VLDL_1$ and $VLDL_2$-TAG pools were calculated from $VLDL_1$ and $VLDL_2$-TAG concentration and plasma volume which was determined by the method of Pearson et al [2].

Kinetic modelling of apoB

$VLDL_1$, $VLDL_2$, IDL, LDL_2 and LDL_3 apoB FCR and production rate were determined using a multi-compartmental model using SAAM II software which incorporated a forcing function corresponding to precursor (α-KIC) enrichment and a delay function accounting for the amount of time required for synthesis and production rate of $VLDL_1$ and $VLDL_2$-apoB. Similar to the TAG model, a delay compartment consisting of a five-compartment chain was added to account for time required for the synthesis and secretion of VLDL1 and VLDL2-apoB. The model is schematically depicted in Figure 3. Production rate (mg/day) was calculated as the product of FCR and the apoB pool size. ApoB pool size (mg) was calculated as the product of apoB concentration and plasma volume (determined as described above)

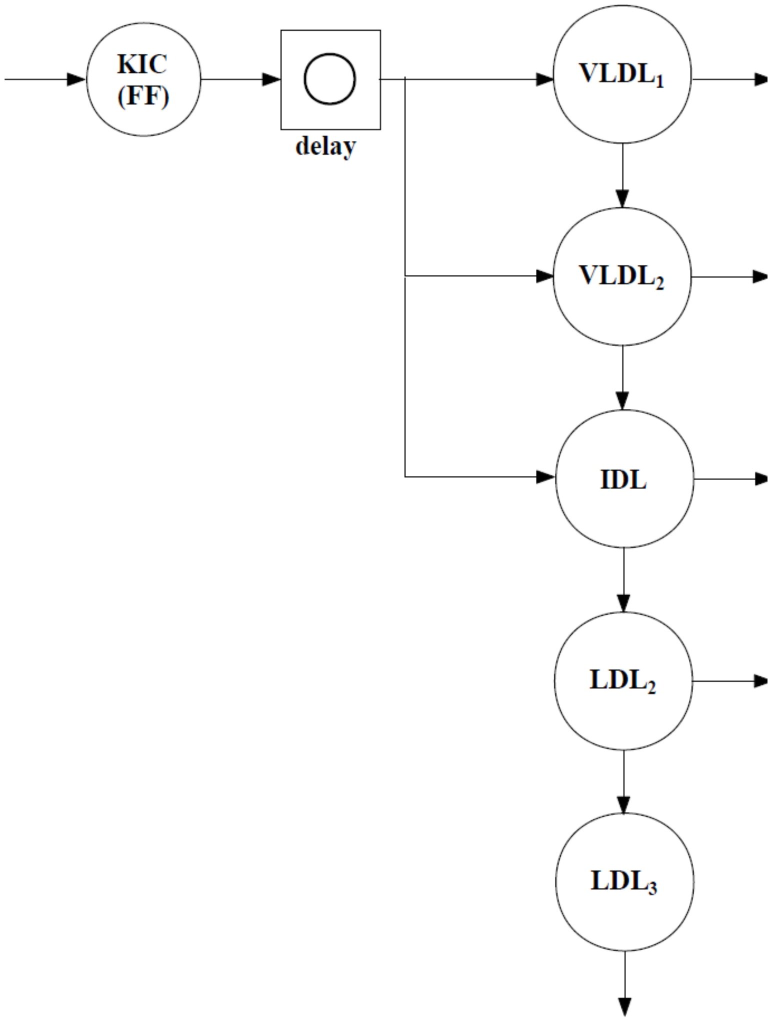

Figure 3. Schematic description of the model used to describe TTRs of $VLDL_1$ $VLDL_2$, IDL, LDL_2 and LDL_3-apoB.

In both models, the parameters were estimated using the weighted non-linear regression analysis. The weights were reciprocal to the variance of the measurement error. The measurement error was assumed uncorrelated with zero mean; a constant standard deviation of 0.005% below TTR of 0.1% and a constant coefficient of variation of 5% above TTR of 0.1%.

References

1. Diraison, F., Pachiaudi, C. and Beylot, M. (1996) In vivo measurement of plasma cholesterol and fatty acid synthesis with deuterated water: determination of the average number of deuterium atoms incorporated. Metabolism **45**, 817-821

2. Diraison, F. Pachiaudi, C. Beylot, M. (1997) Measuring lipogenesis and cholesterol synthesis in humans with deuterated water: use of simple gas chromatographic/mass spectrometric techniques. J. Mass Spectrom. **32**, 81-86.

3. Pearson, T.C., Guthrie, D.L., Simpson, J., Chinn, S., Barosi, G., Ferrant, A., Lewis S.M. and Najean, Y. (1995) Interpretation of measured red cell mass and plasma volume in adults: Expert Panel on Radionuclides of the International Council for Standardization in Haematology. Br. J. Haematol.**89**, 748-756

Table 1. Intake of energy and macronutrients

	NAFLD (n=11)		Controls (n=14)	
	High sugar	Low sugar	High sugar	Low sugar
Total energy MJ/d	10.6±8.6	9.6±6.2	10.6±5.5	10.1±4.3
Carbohydrate g/d	311±22	240±14	342±20	270±18
% energy	50±2	42±2[a]	54±2	44±2[b]
Total sugars g/d	168±15	53±6[b]	177±14	58±6[b]
% energy	27±2	9±1[b]	28±2	10±1[b]
Starch g/d	143±10	187±9[a]	165±11	212±14[a]
% energy	23±1	33±2[b]	26±1	35±1[b]
NMES g/d	152±13	31±2[b]	164±15	33±4[b]
% energy	25±2	6±0.4	26±2	5±0.5
Protein g/d	92±9	98±10	92±5	96±5
% energy	15±1	17±1	15±1	16±1
Total fat g/d	81±13	86±10	75±6	92±5
% energy	28±3	33±2	26±2	34±1[b]
SFA g/d	34±6	36±6	27±3	35±3
Fibre g/d	21±2	23±3	21±2	25±2
Sodium g/d	3.0±0.4	3.5±0.3	2.8±0.2	3.9±0.3[b]

Values are means ± SEM. high sugar versus low sugar [a]P <0.01, [b]P <0.001.

Table 2. Body fat distribution measured by MRS

	NAFLD (n=7)		Controls (n=10)	
	High sugar	Low sugar	High sugar	Low sugar
Total body fat (kg)	26.6±1.5	24.7±1.0	24.8±1.9	23.2±2.5
Total Subcutaneous fat (kg)	17.6±0.8	16.7±0.8	17.6±1.7	16.6±1.8
Total internal fat (kg)	9.0±0.9	8.0±0.5	7.3±0.5	6.6±0.8
Abdominal sub-cutaneous fat (kg)	5.2±0.5	4.8±0.3	5.2±0.7	4.8±0.7
Peripheral sub-cutaneous fat (kg)	12.4±0.6	11.9±0.3	12.4±1.0	11.8±1.2
Visceral fat (kg)	4.6±0.4	4.8±0.4	4.0±0.3	3.6±0.5
Non-visceral internal fat (kg)	4.4±0.9	3.2±0.2	3.3±0.3	3.0±0.3

Values are mean ± SEM

Figure S1

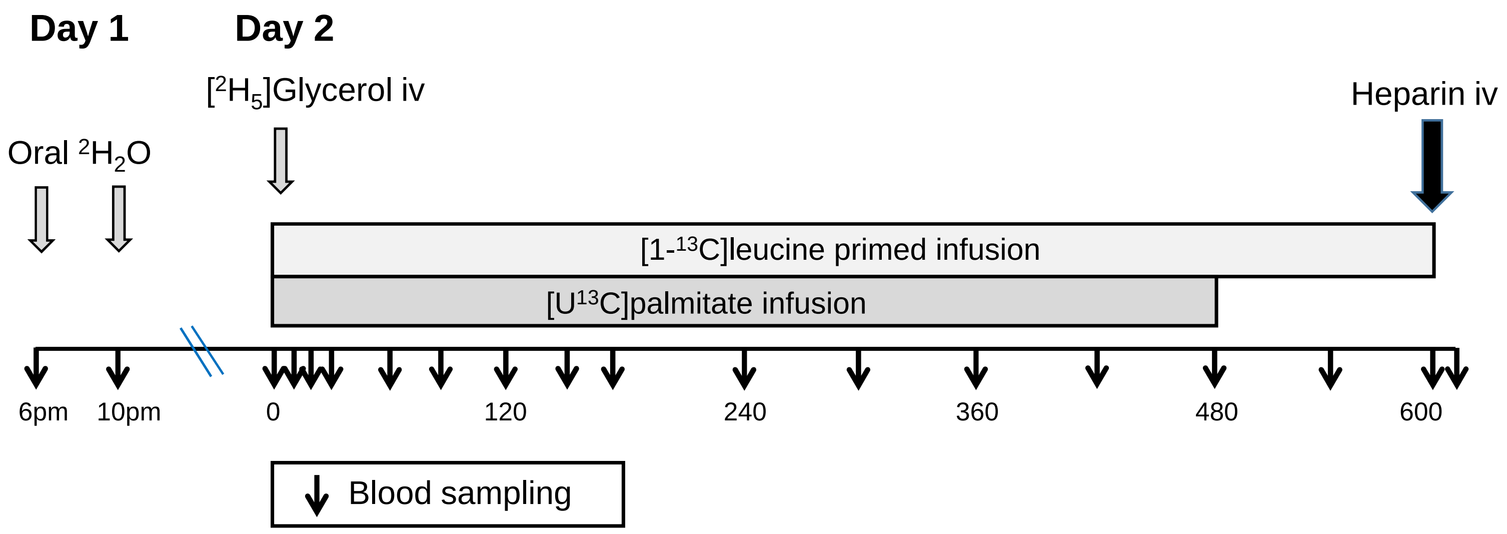

Day 1
Day 2
[2H5]Glycerol iv
Heparin iv
Oral 2H2O
[1-13C]leucine primed infusion
[U13C]palmitate infusion
6pm
10pm
0
120
240
360
480
600
Blood sampling

Figure S2

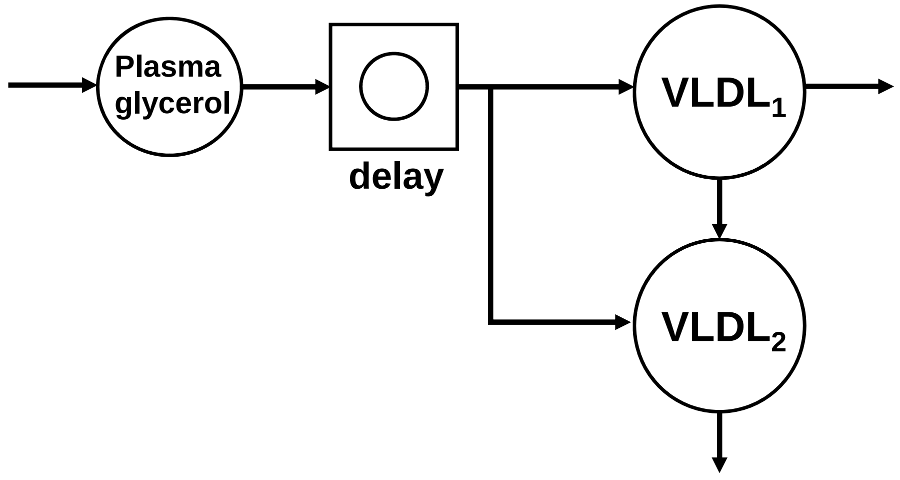

Figure S3

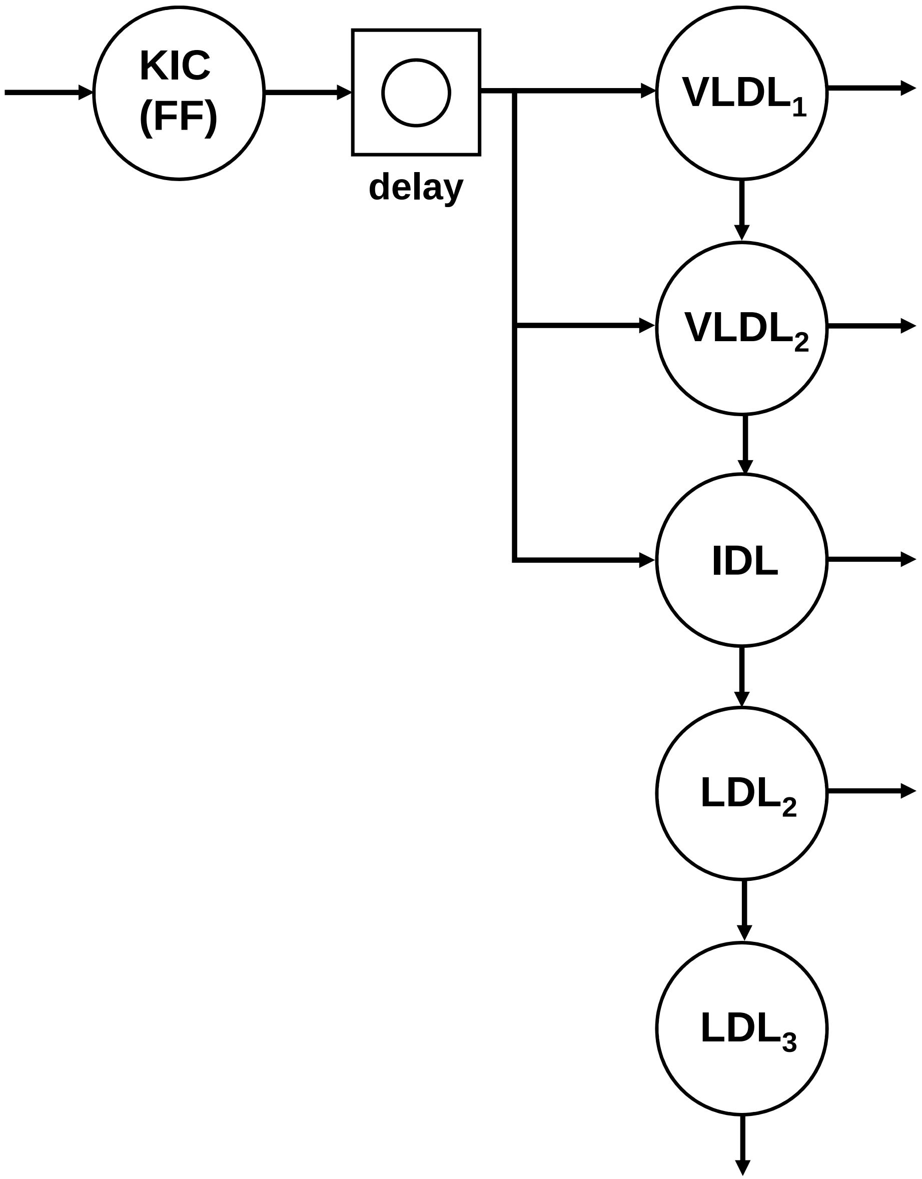

Supplement: Supplementary file 1 [file cs-131-cs20171208_supp1.pdf]
